# PyLipID: A Python Package for Analysis of Protein–Lipid Interactions from Molecular Dynamics Simulations

**DOI:** 10.1021/acs.jctc.1c00708

**Published:** 2022-01-12

**Authors:** Wanling Song, Robin A. Corey, T. Bertie Ansell, C. Keith Cassidy, Michael R. Horrell, Anna L. Duncan, Phillip J. Stansfeld, Mark S. P. Sansom

**Affiliations:** † Department of Biochemistry, University of Oxford, South Parks Road, Oxford OX1 3QU, United Kingdom; ‡ Rahko, Clifton House, 46 Clifton Terrace, Finsbury Park, London N4 3JP, United Kingdom; § School of Life Sciences & Department of Chemistry, University of Warwick, Coventry CV4 7AL, United Kingdom

## Abstract

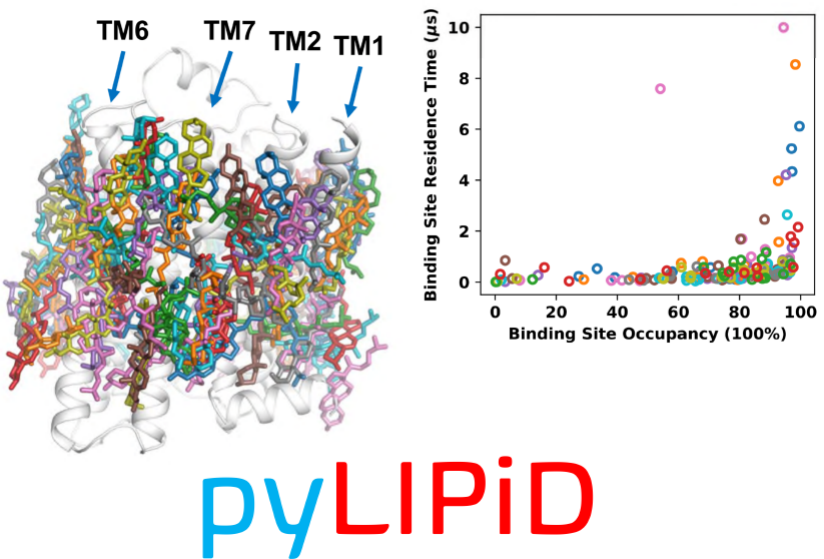

Lipids play important modulatory and structural roles for membrane proteins. Molecular dynamics simulations are frequently used to provide insights into the nature of these protein–lipid interactions. Systematic comparative analysis requires tools that provide algorithms for objective assessment of such interactions. We introduce PyLipID, a Python package for the identification and characterization of specific lipid interactions and binding sites on membrane proteins from molecular dynamics simulations. PyLipID uses a community analysis approach for binding site detection, calculating lipid residence times for both the individual protein residues and the detected binding sites. To assist structural analysis, PyLipID produces representative bound lipid poses from simulation data, using a density-based scoring function. To estimate residue contacts robustly, PyLipID uses a dual-cutoff scheme to differentiate between lipid conformational rearrangements while bound from full dissociation events. In addition to the characterization of protein–lipid interactions, PyLipID is applicable to analysis of the interactions of membrane proteins with other ligands. By combining automated analysis, efficient algorithms, and open-source distribution, PyLipID facilitates the systematic analysis of lipid interactions from large simulation data sets of multiple species of membrane proteins.

## Introduction

Cell membranes typically contain hundreds of different lipid species, asymmetrically distributed between two membrane leaflets.^1,2^ These lipid molecules are locally organized into lateral domains of distinct composition.^3,4^ The combination of these various chemical structures and microdomains results in a diverse lipid landscape that is fully exploited by membrane proteins, especially those involved in cellular signaling.^5,6^ The regulatory roles of membrane lipids include ion channel activation^7,8^ and allosteric modulation of G-Protein Coupled Receptors (GPCRs) and other receptors.^9−13^ Lipid molecules may also strengthen domain and/or subunit interactions in more complex membrane proteins.^14,15^ It is therefore of importance to characterize protein–lipid interactions in order to reach an understanding of the dynamics and functions of membrane proteins.

A number of biophysical techniques can reveal the presence of protein–lipid interactions; e.g., see refs (16 and 17). In particular, following recent advances in single-particle cryo-EM^18^ including the use of nanodiscs to preserve a lipid bilayer environment,^19^ increasing numbers of membrane proteins structures have been determined at near atomic resolution with bound lipids present in the structures.^20^ These membrane protein structures provide gateways for understanding how lipids may modulate protein function but also pose challenges regarding the identification of interacting lipid species.

Computational approaches, especially molecular dynamics (MD) simulations, have played an increasingly important role as a high throughput “computational microscope”^21^ for the identification of protein–lipid interactions. Thanks to ongoing increases in computer power, development of improved atomistic and coarse-grained force fields,^22^ and development of tools to automate setup of membrane simulations,^23,24^ MD simulations have been applied to many membrane proteins and lipids, providing invaluable structural and mechanistic insights into their protein–lipid interactions.^25−28^ While there is not space here to extensively review simulation studies of membrane protein–lipid interactions, one might recall pioneering studies including the interaction of polyunsaturated fatty acids and cholesterol with rhodopsin,^29−31^ the interaction of cholesterol and other lipids with class A GPCRs,^32−34^ and more recently development of a range of methods for estimation of the free energy of interactions of lipids with membrane proteins via simulation.^35−37^

For the study of protein–lipid interactions, CG force fields can explore lipid binding sites in an unbiased fashion with sufficient sampling due to the decreased degrees of freedom of the underlying model. The Martini force field^38−40^ is widely used for biomembrane applications. Simulations using Martini have identified lipid binding sites on a range of proteins^41−43^ and have assisted the interpretation of lipid-like density in cryo-EM maps.^44^ Some simulation studies have adopted a serial multiscale approach in which CG simulations are used to probe the lipid binding sites and bound lipid identities followed by atomistic simulations to study residue-level protein–lipid interactions. The conversion of Martini models to atomistic ones can be achieved by resolution back-mapping tools.^45−50^

With the increasing number of membrane protein structures determined at high resolution by cryo-EM and the increasing complexity of simulated membranes, the use of MD simulations to study protein–lipid interactions is accompanied by two challenges:

(1) How to automatically determine lipid binding sites from simulations? Some simulation studies have used the average lipid density to approximately locate lipid binding sites and subsequently manually assigned bound poses. Such an approach includes an element of subjectivity and may be a bottleneck for large scale comparative simulations. So, can we determine the lipid binding sites automatically via a statistically robust method? Additionally, can we systematically produce representative bound poses from the trajectories for further analysis?
(2) How to optimally quantify and characterize lipid interactions? Simulation studies have used, e.g., average lipid occupancies or the fraction of trajectory frames in which lipid contacts are formed to a given residue to measure lipid interactions. Can we rigorously calculate lipid interactions with binding sites in addition to individual residues to allow for more direct comparison with experiments?

To provide a unified solution to the above-mentioned problems, we have developed a Python package, PyLipID, to assist analysis of protein–lipid interactions from MD simulations. PyLipID identifies binding sites by calculating the community structures in the interaction network of protein residues. This method was initially applied to the analysis of cholesterol^51^ and other lipid^43^ binding sites on the Kir2.2 channel. On the basis of the identified binding sites, PyLipID can find representative bound poses for each site. This is achieved by evaluating all the bound poses using an empirical scoring function of the lipid density in the chosen binding site. This functionality allows for further structural analysis of the protein–lipid interactions and makes it possible to automate pipelines for converting bound lipids poses in CG models into atomistic ones for use in multiscale simulation studies. PyLipID can also cluster the bound poses for binding sites to provide a more in-depth analysis of the lipid interactions. To describe lipid interactions, PyLipID calculates residence times, in addition to other commonly used metrics such as averaged interaction duration, lipid occupancy, and the average number of surrounding lipids, for both individual protein residues and the calculated binding sites. The calculation of residence times reveals the dynamical behavior of bound lipids, and calculations based on binding sites allow for improved characterization of the binding events. Notably, PyLipID uses a dual-cutoff scheme to deal with the “rattling in a cage” effect sometimes seen in protein–lipid simulations.

In the following sections, we first introduce the methodological details of PyLipID. Then, we illustrate the PyLipID analysis pipeline using cholesterol binding to a panel of GPCRs as an example. Subsequently, we present two cases of the application of PyLipID to interactions of membrane proteins with phospholipids illustrating the potential application of PyLipID to assist the interpretation of lipid-like densities in cryo-EM maps. Finally, we demonstrate the application of PyLipID to nonlipid molecules, using it to characterize ethanol binding to the cytoplasmic domain of the *Bacillus subtilis* (*B. subtilis*) McpB chemoreceptor as seen in atomistic simulations.^52^

## Methods

PyLipID is an open-source package available on GitHub (https://github.com/wlsong/PyLipID). The documentation and tutorials can be found at the ReadtheDocs server https://pylipid.readthedocs.io. A tutorial script that runs the PyLipID analysis can be found at the documentation Web site.

### Overview of Code

**Figure 1:**
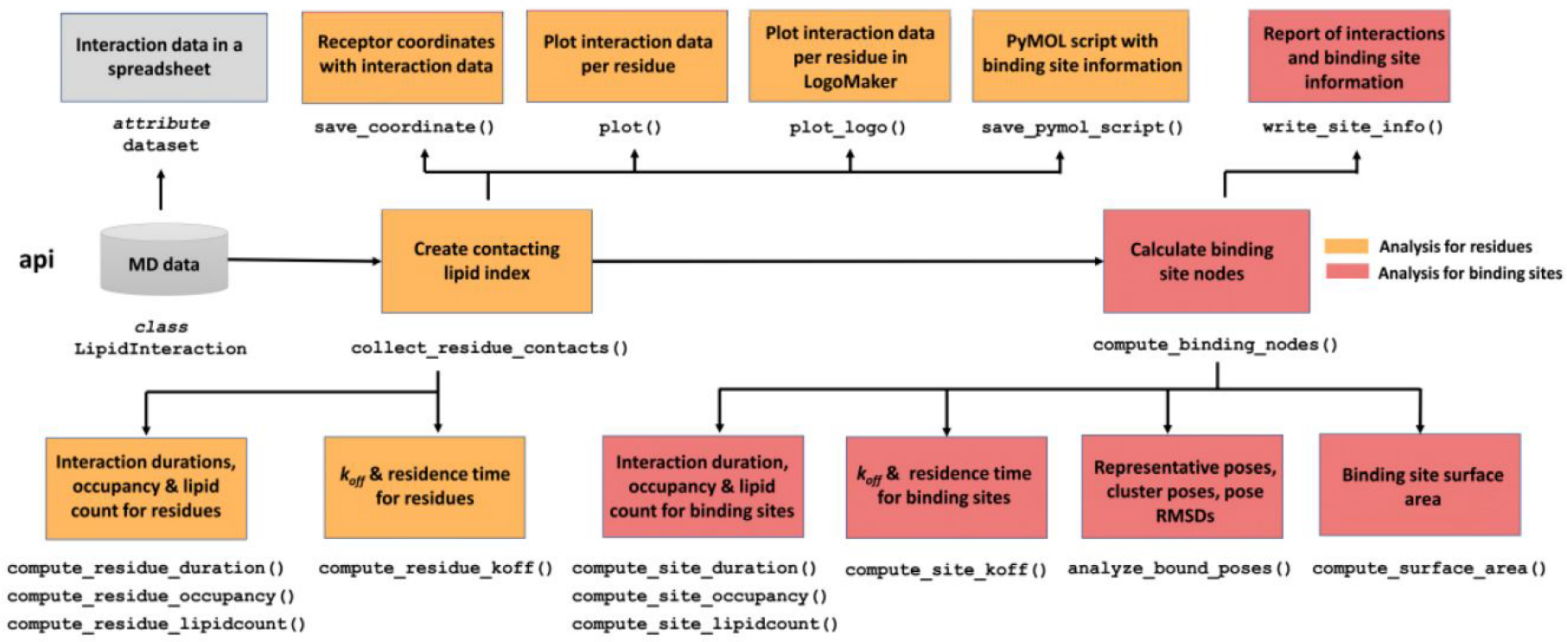
PyLipID package design: api module structure. api is the outer layer module and its main class LipidInteraction handles the analysis workflow. The class object LipidInteraction loads the trajectory data, and the methods of this class object carries out the analysis for protein residues (yellow boxes) and for binding sites (red boxes). This class object also has an attribute dataset, which is a spreadsheet object storing interaction data and allows for further manipulation. PyLipID has another three modules, func, plot, and util, which provide functions for doing the computationally intensive analysis, as used by LipidInteraction (SI Figure S1).

The current PyLipID package contains four modules: api, func, plot, and util. api is the outer layer module that handles the analysis workflow and provides some convenient functions for plotting and saving data (Figure 1), whereas the remaining modules provide functions that are deployed by api for the heavy lifting in the analysis (Supporting Information (SI) Figure S1). Such a structure allows for extension of PyLipID functionalities with minimal changes to the code base. PyLipID reports results in various forms.

#### api

This module contains the main Python class LipidInteraction that reads trajectory information, analyzes lipid interactions, and writes/plots interaction data. The PyLipID analyses are carried out by the class methods of LipidInteraction, which can be divided into two groups: methods for analysis of interactions with protein residues and with the calculated binding sites. Each group has a core function to collect/calculate the required data for the rest of the functions in that segment, i.e., collect_residue_contacts() that builds a lipid index for residues as a function of time for residue analysis and compute_binding_nodes() that calculates the binding sites using the interaction correlation matrix of the residues. The remainder of the methods in each group are independent of each other and calculate different properties of lipid interactions and of the binding site. LipidInteraction also has an attribute dataset which stores the calculated interaction data in a spreadsheet as a pandas.DataFrame, and updates automatically after each of the calculations. It records interaction data for protein residues by row, including interaction residence times, averaged durations, occupancy, and lipid count, and the associated interaction data for the binding site to which the residue belongs. This pandas.DataFrame data structure allows for convenient checking of the interaction data and provides users with maximum flexibility to further process PyLipID outputs. For the computationally intensive functions, e.g., calculation of *k*_off_, bound poses or binding site surface areas, PyLipID uses a Python multiprocessing library to speed up the calculations. Users can specify the number of CPUs these functions can use; otherwise, all available CPUs will be used by default.

#### func

This module comprises the following four submodules: interaction that contains functions for calculation of continuous contacts using a double-cutoff scheme; kinetics for calculation of *k*_off_ and residence time; binding_site for calculation of binding sites using the Louvain method^53^ as well as the analysis of bound poses and surface area; and clusterer for clustering the bound poses.

#### plot

This module provides convenient functions to visualize the interaction data, e.g., plots of *k*_off_, interaction as a function of residue index, the correlation matrix of lipid interactions for residues, and binding site data.

#### util

This is the location of housekeeping functions. For example, trajectory contains functions for obtaining topology information from trajectories.

### Technical Features

PyLipID is written in Python and compatible with versions 3.6+. It uses MDtraj^54^ to handle trajectories and coordinates, and thus it is compatible with all major simulation packages. PyLipID reads the molecule topology from trajectories and uses a distance-based method to measure contacts; it is therefore applicable to the calculation of binding characteristics for any type of molecule. In the following section, we will introduce the technical features of PyLipID and their implementation in the code.

#### Lipid Topology

The lipid topology information is read from trajectories, and contacts are calculated on the basis of the minimum distance of the lipid molecule to the protein. A lipid molecule is considered as being in contact with a residue when the distance of any atoms of the lipid molecule to any atoms of the residue is smaller than the provided distance cutoff. PyLipID also allows for selection of lipid atoms used for defining contacts. This option can be useful for cases in which excluding some atoms (e.g., the tails of phospholipids) could generate improved definition of binding sites. Given how lipid contact is calculated, PyLipID does not need to store or define any lipid topology information in the code, which allows PyLipID to calculate the contact of any kind of object with a protein on the basis of their distances.

#### Dual-Cutoff Scheme

Due to the smoothened potentials and/or shallow binding pockets, CG simulations may show a “rattling in a cage” effect, in which lipid molecules undergo rapid changes in protein contacts without full dissociation from a given site, such that the minimum distances between the two contacting objects may experience sudden jumps. This is to be expected when using a single cutoff to define a boundary between two states that do not have a simple clear barrier between them. For example, it has also been observed in atomistic simulations of loosely bound cholesterol molecules.^55^ To deal with these frequently encountered rapid fluctuations in the bound pose, PyLipID adopts a dual-cutoff scheme, which uses a lower and upper distance cutoff to measure the status of contact (SI Figure S2). The duration of a continuous contact is determined from the time point when a molecule moves closer than the lower distance cutoff until the time point when the molecule moves beyond the upper cutoff distance. The SI provides a more detailed discussion of cutoff values and their impact on binding site calculations (Section S1 and Figures S3–S9). In addition to the contact duration, PyLipID provides three other metrics for characterization of lipid contacts: lipid duration, which is the average duration of the collected contacts; lipid occupancy, which is the percentage of frames in which any lipid contact is formed; and lipid count, which is the number of surrounding molecules of the specified lipid species. Both lipid occupancy and lipid count are calculated using the lower distance cutoff.

#### Residence Time

**Figure 2:**
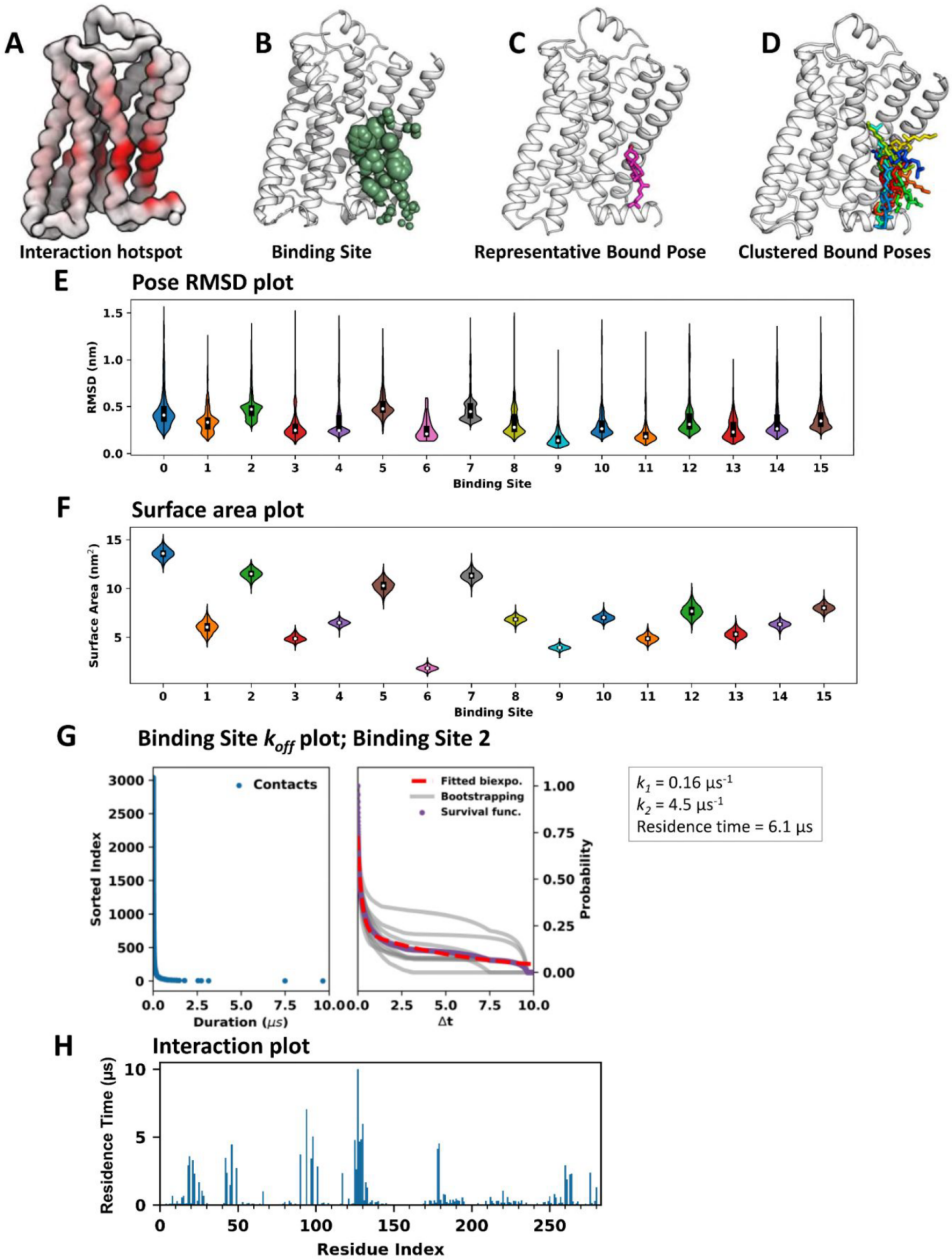
Illustration of PyLipID analysis outputs, using simulations of the β_2_AdR in the presence of cholesterol as an example. PyLipID can save interaction data in the B-factor column of a PDB file of the protein coordinates using save_coordinate(). Such a coordinate file can be loaded into visualization software and colored on the basis of B-factor to show the interaction hotspot (A). PyLipID can generate a Python script that maps the binding site information to a receptor structure in a PyMOL session, in which residues from the same binding site are shown in spheres in the same color and the sphere scales correspond to their interaction with the lipid. This is accomplished by save_pymol_script() (B). The method of analyze_bound_pose() can find the representative bound pose for a binding site (C), and cluster all the bound poses in a binding site (D). This method can also calculate the RMSDs of the bound poses for a binding site and provide a convenient plot of the RMSDs (E). The method compute_surface_area() calculates binding site surface area as a function of time and plots the surface area data (F). PyLipID calculates interaction residence times for residues using compute_residue_koff() and for binding sites using compute_site_koff(). Both methods generate *k*_off_ plots, in which the durations of the collected contacts are plotted in a sorted order in the left panel and the normalized survival function together with the fitted data are plotted in the right panel (G). The plot() method can draw the interaction data as a function of residue index (H).

The residence time provides useful insights^56^ into the dynamic behavior of bound lipids which, due to their interaction with the protein, are no longer diffusive.^41,43^ Indeed, both prolonged interactions and transient contacts are observed for lipids on the protein surface. The residence time, which is calculated from a survival time correlation function, describes the relaxation of the bound lipids and can be divided into long and short decay periods, which correspond to specific interactions and transient contacts, respectively. PyLipID calculates the survival time correlation function σ(*t*) as follows:
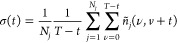
where *T* is the length of the simulation trajectory, *N*_*j*_ is the total number of lipid contacts, and ∑_ν=0_^*T*–*t*^*ñ*_*j*_(*v*, *v* + *t*) is a binary function that takes the value 1 if the contact of lipid *j* lasts from time ν to time ν + *t* and 0 otherwise. The values of σ(*t*) are calculated for every value of *t* from 0 to *T* ns, for each time step of the trajectories, and normalized by dividing by σ(0), so that the survival time-correlation function has value 1 at *t* = 0. The normalized survival function is then fitted to a biexponential to model the long and short decays of lipid relaxation, respectively:
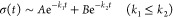
PyLipID stores the fitting parameters for both exponential components, reporting those for the slower decay. Thus, PyLipID takes *k*_1_ as the dissociation constant, *k*_off_, and calculates the residence time from τ = 1/*k*_off_. PyLipID measures the *r*^2^ of the biexponential fitting to the survival function to show the quality of the *k*_off_/residence time estimation. In addition, PyLipID bootstraps the contact durations and measures the *k*_off_ /residence time of the bootstrapped data, to report how well lipid contacts are sampled from simulations. The lipid contact sampling, the curve-fitting, and the bootstrap results can be conveniently checked for individual residues and the calculated binding sites via the *k*_off_ plots generated by PyLipID (see Figure 2 and discussion below for further details).

#### Calculation of Binding Sites

Binding sites are defined on the basis of a community analysis of protein residue-interaction networks that are created from the lipid-interaction correlation matrix. Given the basic definition of a lipid binding site, namely, a cluster of residues that bind to the same lipid molecule at the same time, PyLipID creates a distance vector that records the distances to all lipid molecules as a function of time for each residue and constructs a lipid-interaction network in which the nodes are the protein residues and the weights are the Pearson correlation coefficients of pairs of residues that are calculated from their distance vectors (SI Figure S10). PyLipID then decomposes this interaction network into subunits or communities, which are groups of nodes that are more densely connected internally than with the rest of the network. For the calculation of communities, PyLipID uses the Louvain algorithm^53^ that finds high modularity network partitions effectively. Modularity, which measures the quality of network partitions, is defined as^57^
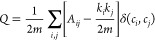
where *A*_*ij*_ is the weight of the edge between node *i* and node *j*; *k*_*i*_ is the sum of weights of the nodes attached to the node *i*, i.e., the degree of node; *c*_*i*_ is the community to which node *i* is assigned; δ(*c*_*i*_,*c*_*j*_) is 1 if *i* = *j* and 0 otherwise; and , i.e., the number edges. In the modularity optimization, the Louvain algorithm orders the nodes in the network and, then, one by one, removes and inserts each node in a different community *c*_*i*_ until there is no significant increase in modularity. After modularity optimization, all the nodes that belong to the same community are merged into a single node, of which the edge weights are the sum of the weights of the comprising nodes. This optimization–aggregation loop is iterated until all nodes are collapsed into one. PyLipID allows for filtering of the communities on the basis of their sizes, i.e., filtering the binding sites on the basis of the number of comprising residues. By default, PyLipID returns binding sites of at least four residues. This filtering step is particularly helpful for analysis of a small number of trajectory frames, in which false correlation is more likely to happen among two or three residues. The output from this calculation is a list of binding sites containing sets of binding site residue indices.

#### Calculation of Representative Bound Poses

PyLipID evaluates bound poses using an empirical density-based scoring function and writes out the most sampled bound poses for each binding site. The scoring function of a lipid pose at a binding site is defined as
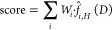
where *W*_*i*_ is the weight given to atom *i* of the lipid molecule, *H* is the bandwidth, and *f̂*_*i*,*H*_(*D*) is a multivariate kernel density estimation of the position of atom *i* based on the positions of all bound lipid poses in that binding site. The position of atom *i* is a *p*-variant vector, [*D*_*i*__1_,*D*_*i*__2_,...,*D*_*ip*_], where *D*_*ip*_ is the minimum distance to the residue *p* of the binding site. PyLipID uses the Gaussian kernel function and, by default, a bandwidth of 0.15. The multivariant kernel density estimation is implemented by *statsmodels*.^58^ Higher weights can be given to, e.g., the headgroup atoms of phospholipids, to generate better defined binding poses, but all lipid atoms are weighted equally by default. In the density estimation, PyLipID uses the relative positions of lipid atoms in the binding site, which makes the analysis of a binding site independent of local protein conformational changes. Lipid poses with the highest scores are considered as the representative bound poses for their binding site and can be written out, along with the protein conformation to which it binds, in any format supported by MDTraj (e.g., pdb and gro). See SI Section S1 for more detailed discussion on the choice of cutoff values and representative bound poses/clustered poses.

#### Clustering of Bound Lipid Poses

PyLipID can cluster the bound lipid poses of a binding site into a user-specified number of clusters using KMeans, in a “supervised” fashion or cluster the poses using a density-based cluster, DBSCAN, in an “unsupervised” fashion. In the former case, the KMeans function from *scikit-learn*(59) is used to separate the samples into *n* clusters of equal variances, via minimizing the *inertia*, which is defined as
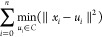
where *u*_*i*_ is the “centroid” of cluster *i*. KMeans scales well with a large data set but performs poorly with clusters of varying sizes and densities, which are often the case for lipid poses in a binding site.

When the number of clusters is not provided by the user, PyLipID uses the DBSCAN algorithm implemented in *scikit-learn* to find clusters of core samples of high density. A sample point *p* is a core sample if at least *min_samples* points are within distance ε of it. A cluster is defined as a set of sample points that are mutually density-connected and density-reachable; i.e., there is a path ⟨*p*_1_,*p*_2_,...,*p*_*n*_⟩ where each *p*_*i*+1_ is within distance ε of *p*_*i*_ for any two *p* in the set. The values of *min_samples* and ε determine the performance of this cluster. PyLipID sets the ε as the knee point of the *k*-distance graph. Once ε is set, the clustering results with all possible *min_samples* are checked using the Silhouette coefficient:
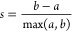
where *a* is the mean distance between a sample and all other points in the same cluster and *b* is the mean distance between a sample and all other points in the next nearest cluster. The Silhouette coefficient is between −1 and 1, and higher scores suggest better defined clusters. The clustering result with the highest Silhouette score is returned as the optimal clustering results. For writing out the cluster poses, PyLipID randomly selects one pose from each cluster in the case of using KMeans or one from the core samples of each cluster when DBSCAN is used and writes the selected lipid pose with the protein conformation to which it binds using MDTraj. The relative position of lipid poses in the binding site, i.e., [*D*_1_,*D*_2_,...,*D*_*i*_], where *D*_*i*_ is the distance vector of atom *i* to the residues in the binding site, is used as the pose coordinates for clustering. Principal component analysis is used to decrease the lipid coordinate dimension before the clustering.

#### Calculation of Pose RMSD

The root-mean-square deviation (RMSD) of a lipid bound pose in a binding site is calculated from the relative position of the pose in the binding site compared to the average position of the bound poses. Thus, the pose RMSD is defined as
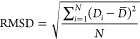
where *D*_*i*_ is the distance vector of atom *i* to the residues in the binding site, *D̅* is the average of the distance vectors of atom *i* from all bound poses in the binding site, and *N* is the number of atoms in the lipid molecule.

#### Calculation of Binding Site Surface Area

The accessible surface area is calculated using the Shrake–Rupley algorithm.^60^ PyLipID strips the protein coordinates out of the simulation system and obtains the accessible surface area of a binding site by summing those of its comprising residues. The surface areas of protein residues are calculated by the shrake_rupley function of MDTraj.

## Results

### PyLipID Analysis Outputs, Illustrated for CG Simulations of the Interactions of the β_2_AdR with Cholesterol

Before describing in detail application cases of PyLipID, we provide a brief overview of PyLipID analysis and outputs (Figure 2). As an example, we use cholesterol interaction with the β_2_AdR (a GPCR). A more detailed account of GPCR–cholesterol interactions is provided in a subsequent section. We carried out PyLipID analysis using simulation data from three repeats. Therefore, the reported durations, occupancies, and lipid counts, for both residues and binding sites, by PyLipID were averaged over the repeats and the residence times were calculated from the durations of lipid contacts collected from all repeats. We also recommend evaluating the impact of different dual cutoffs on binding sites and interaction durations, prior to using PyLipID, to find the optimal values (see SI Section S1 and Figures S4–S9). For the case of analysis of cholesterol interactions with GPCRs, we chose to use 0.475 and 0.80 nm for the dual cutoffs.

PyLipID outputs result in different forms to assist different analyses. Each analysis is carried out by a method of the class LipidInteraction. Users may select specific analysis to implement or use the demonstration script provided on the PyLipID Web site to run all of the analysis once. We first calculated cholesterol interaction, i.e., interaction residence times in this case, with receptor residues via the method compute_residue_koff(). To visualize the residue-wise interactions, we used the method save_coordinate() to generate a Protein Databank (PDB) file of the receptor coordinates in which the interaction data are saved in the B-factor column, enabling us to check the locations of interaction hotspots (Figure 2A).

We then calculated the binding sites using the method compute_binding_nodes(). After this step, the cholesterol interactions, i.e., residence times in this case, with these binding sites were calculated using compute_site_koff(). To assist the visualization of these binding sites, we used the method save_pymol_script() to generate a Python script that maps the *binding site* information to receptor structure in a PyMOL session, in which residues from the same binding site are shown as spheres in the same color and the sphere scales correspond to their interactions with the lipid (Figure 2B). This binding site visualization, combined with a binding site summary that was generated by write_site_info(), helped to filter through binding sites and find ones of interest. To analyze the structural details of cholesterol interactions, we used analyze_bound_pose() to find the representative bound pose for a given binding site (Figure 2C) and to cluster all the bound poses in a binding site (Figure 2D). In addition, we also calculated other properties of the binding sites/bound poses, including the RMSDs of bound poses via analyze_bound_poses() (Figure 2E) and the surface areas of the binding site via compute_surface_area() (Figure 2F).

Importantly, when calculating the residence times using either compute_residue_koff() or compute_site_koff(), PyLipID can also generate the *k*_off_ plots, in which the durations of the collected contacts are plotted in a sorted order along with the normalized survival function, fitted biexponential curve, and bootstrapped data (Figure 2G). The quality of the sampling of binding events, which can be checked by the bootstrapping data, and the quality of the evaluation of residence times, which can be checked by *r*^2^ of the curve fitting, were checked when we filtered the binding sites.

### Comparative Analysis of Cholesterol Binding Sites on Selected Class A and B GPCRs

The application of PyLipID through Python scripts allows for a high throughput and systematic analysis of large protein–lipid interaction data sets. Here we demonstrate how PyLipID, in conjunction with CG MD simulations, was used to characterize cholesterol binding sites on GPCRs. We performed 3 × 10 μs CG simulations for each of 10 species of GPCR (see SI Section S1 and Table S1 for simulation details), embedded within a membrane containing 35% cholesterol and applied PyLipID analysis to study the cholesterol interactions with these receptors.

Combining the residence time profiles and molecular visualization, we found that cholesterol interactions were consistently found between transmembrane helices. However, the strength of the interactions (measured as residence times) varied depending on the receptor and the interhelical location. We saw stronger cholesterol interactions with β_2_AdR and D3R at locations around, e.g., TM1, TM7, TM3, and TM4, whereas much weaker interactions were seen in, e.g., C–C chemokine receptors and P2Y1 (SI Figures S2 and S3), suggesting the affinities for cholesterol may vary among receptors and sites.

**Figure 3:**
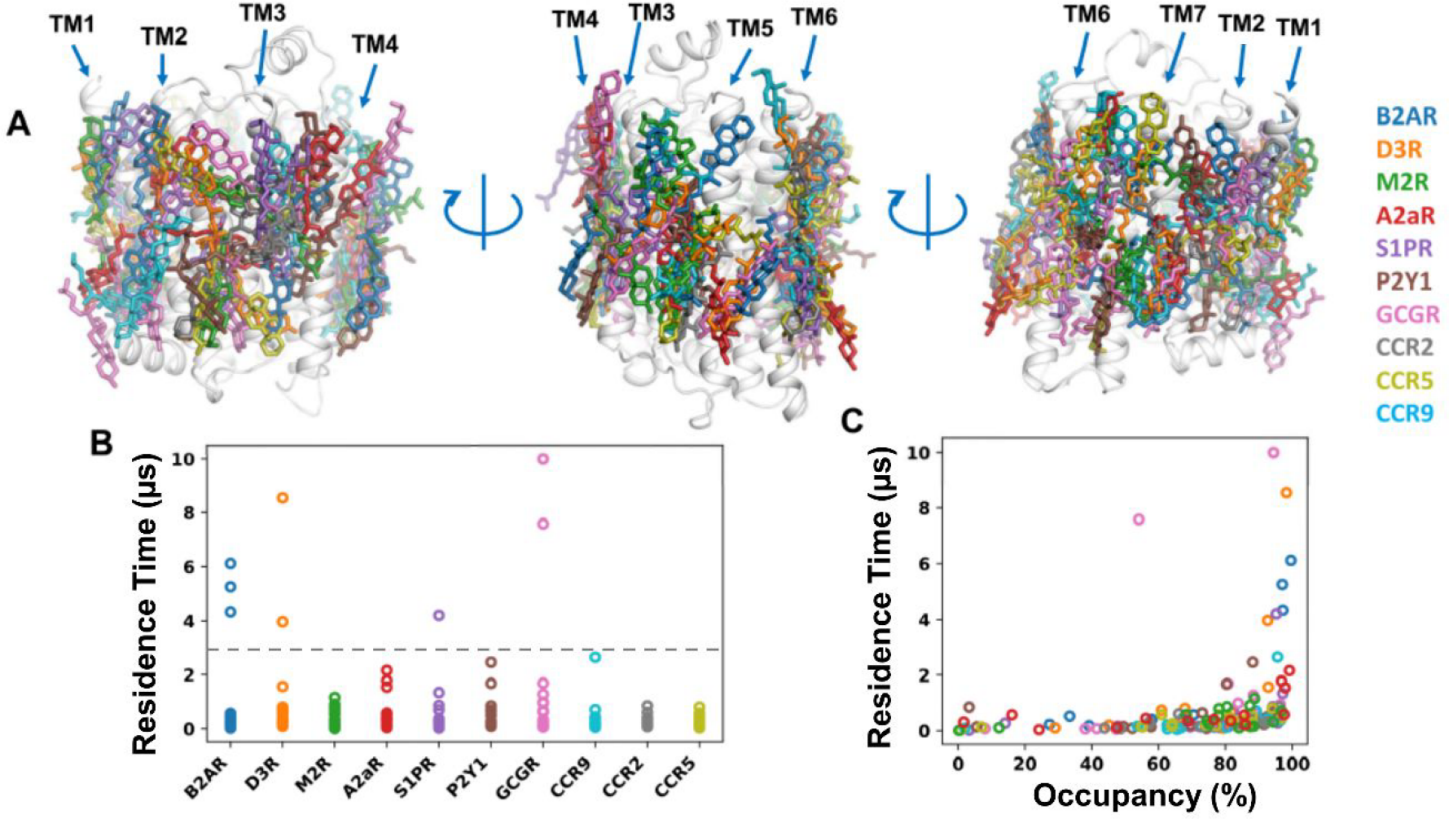
Cholesterol binding sites on GPCRs. (A) Representative cholesterol bound poses of all the binding sites on the 10 GPCRs. The binding sites/poses from all 10 GPCRs are aligned on the β_2_AdR structure. (B) Binding site residence times and (C) binding site occupancy calculated from the 10 GPCRs.

We then analyzed the lipid bound poses in the binding sites. On average, 14–17 cholesterol binding sites were revealed per receptor and, in total, 153 cholesterol binding sites from the 10 receptors. Aligning the representative bound poses from the 10 tested receptors to the β_2_AdR structure revealed that cholesterol molecules can be found in most of the interhelical spaces (Figure 3A). This is in agreement with a recent analysis of the locations of bound cholesterols in GPCR structures, which reports that cholesterol binding sites lack consensus motifs.^61^ This also lends support to the suggested wedge-like role of cholesterols in stabilizing GPCR conformations.^34^

We next calculated the binding site residence times and cholesterol occupancies. Most of the cholesterol binding sites had interaction residence times < 3 μs (Figure 3B). For these sites there was little, if any, correlation between residence time and occupancy (Figure 3C). The high frequency of cholesterol binding and the relatively short residence times suggest that these cholesterol molecules act as annular lipids around GPCRs, forming a cholesterol solvation shell. However, we also detected a number of binding sites with residence times > 3 μs (on β_2_AdR, D3R, S1PR, and GCGR). With one exception these all had an occupancy of >90% (Figure 3C). This suggests that at these sites cholesterol can form longer and more specific interactions.

**Figure 4:**
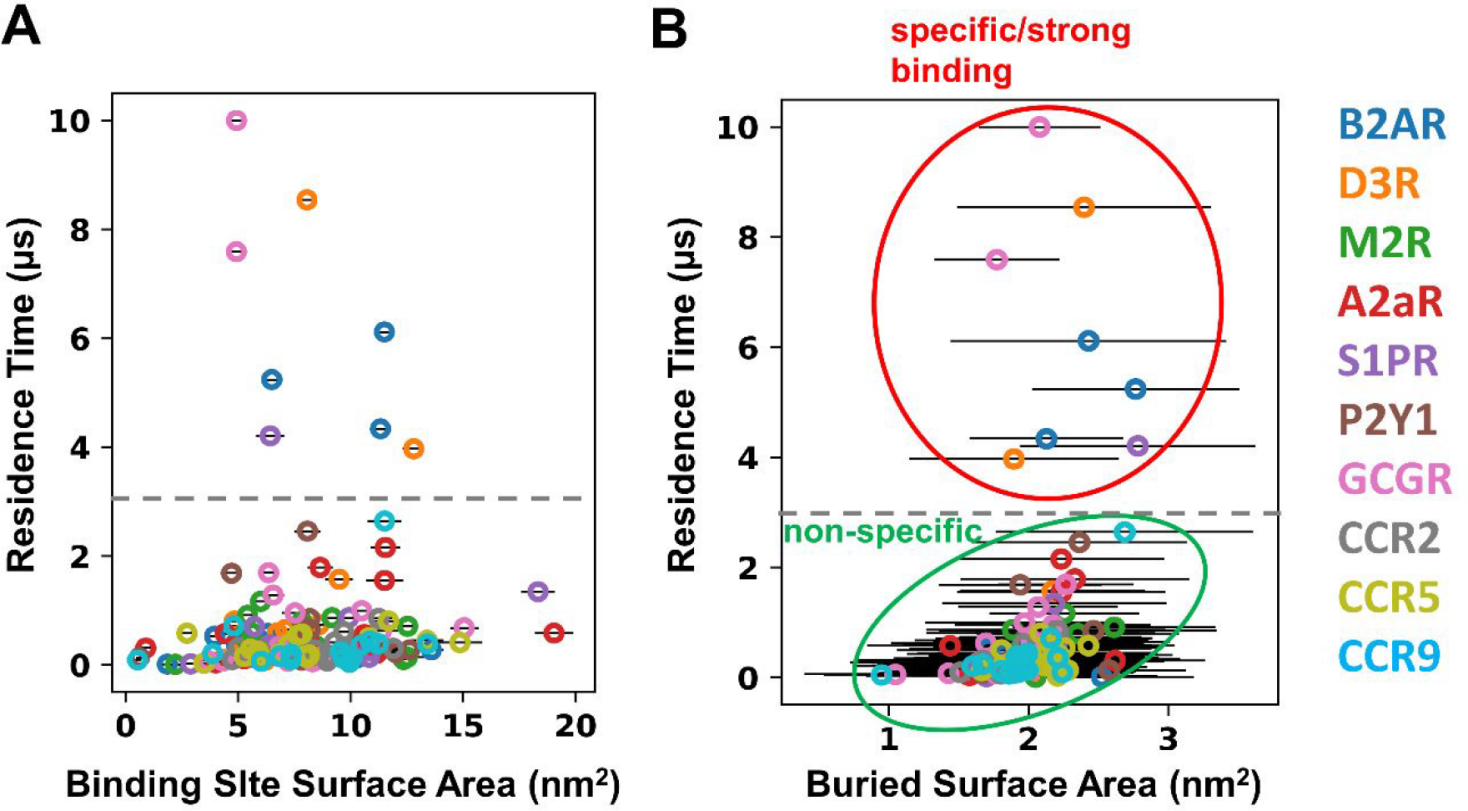
Geometry of cholesterol binding sites on GPCRs. (A) Binding site surface area and (B) the buried surface area of the cholesterol bound in the binding sites on GPCRs. The 3 μs residence time cutoff used to separate nonspecific/annular from specific/tight binding interactions is shown as a gray broken line, and the latter two classes are indicated by the green and red ellipses, respectively, in B.

We then set out to analyze whether there are sequence or structural motifs that determine the length of interaction residence times (i.e., the strength of cholesterol interactions). We first checked whether the size of the binding site affects the interaction. We calculated the binding site surface areas and the buried area, i.e., contacting the surface area of bound cholesterols with the receptor. The stronger cholesterol binding sites (i.e., those with residence time > 3 μs) have mid-range sizes, with surface areas between 5 and 12 nm^2^ (Figure 4A). Visual inspection revealed that the larger binding sites on GPCRs were often flat, shallow, and featureless. The calculation of the buried surface area of cholesterols in the binding sites showed a similar picture, and the bound cholesterols could clearly be separated into two groups (Figure 4B). For the weaker (nonspecific or annular) sites there was perhaps a weak correlation between residence time and buried surface area. For the stronger (specific) sites the contacting surface area did not correlate with the residence times. This suggests that specific binding is more subtly determined than simply the area of the cholesterol binding site on a GPCRs.

**Figure 5:**
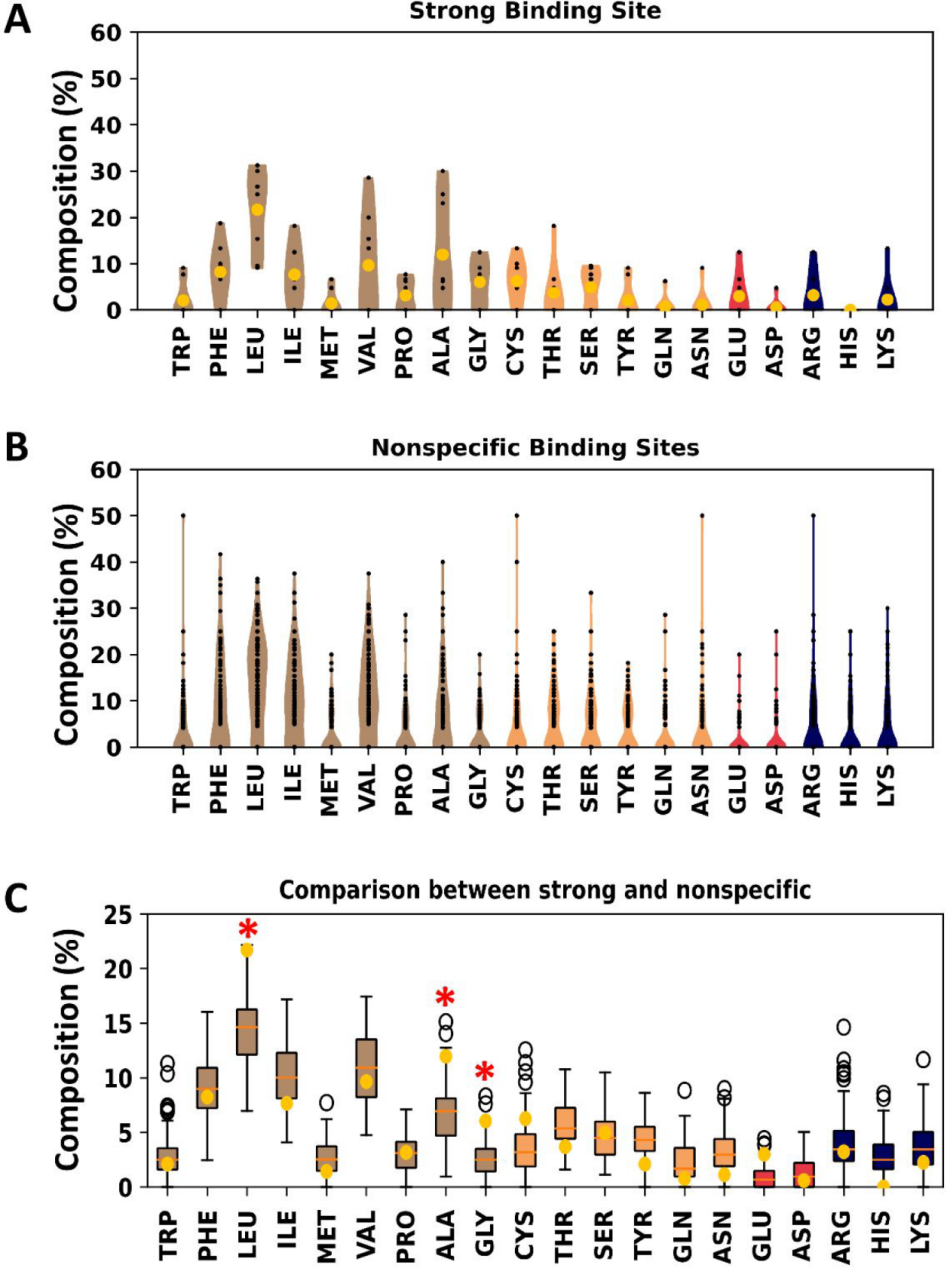
Amino acid composition of cholesterol binding site on GPCRs. (A) Violin plot of the amino acid composition of eight specific binding sites that showed cholesterol residence times longer than 3 μs. (B) Violin plot of the amino acid composition of the 145 binding sites that showed shorter duration cholesterol interactions. (C) Comparison of the binding site amino acid compositions between the bootstrapping values from the 145 nonspecific binding sites (box plot) and the averages from the eight specific binding sites (yellow dot). Data for amino acid compositions are color-coded on the basis of the amino acid chemical property: data for nonpolar amino acids are colored in brown, for polar amino acids in yellow, for acidic amino acids in red, and for basic amino acids in blue. The red asterisks indicate residues where there is a clear difference in composition between the nonspecific and specific site amino acid compositions.

To explore this further, we analyzed the amino acid residue composition of the cholesterol binding sites, looking to see whether longer residence times resulted from a specific composition of the binding sites. We again set a residence time cutoff of 3 μs to separate weaker and strong binding sites, selected from a plot of the sorted residence times (SI Figure S13). We calculated the amino acid composition for each binding site in the two classes (Figure 5A,B). To compare the two sets of data, which have very different sizes, we bootstrapped the data from the nonspecific binding sites. Here, we randomly selected eight binding sites and compared their average amino acid composition to the eight strong binding sites. This comparison revealed that the strong cholesterol binding sites have increased occurrence of Leu, Ala, and Gly residues (Figure 5C). This is broadly consistent with a recent structural analysis,^61^ which failed to reveal distinct sequence motifs for cholesterol binding to GPCRs but which reported cholesterol microenvironments enriched in Leu, Ala, Ile, and Val residues.

**Figure 6:**
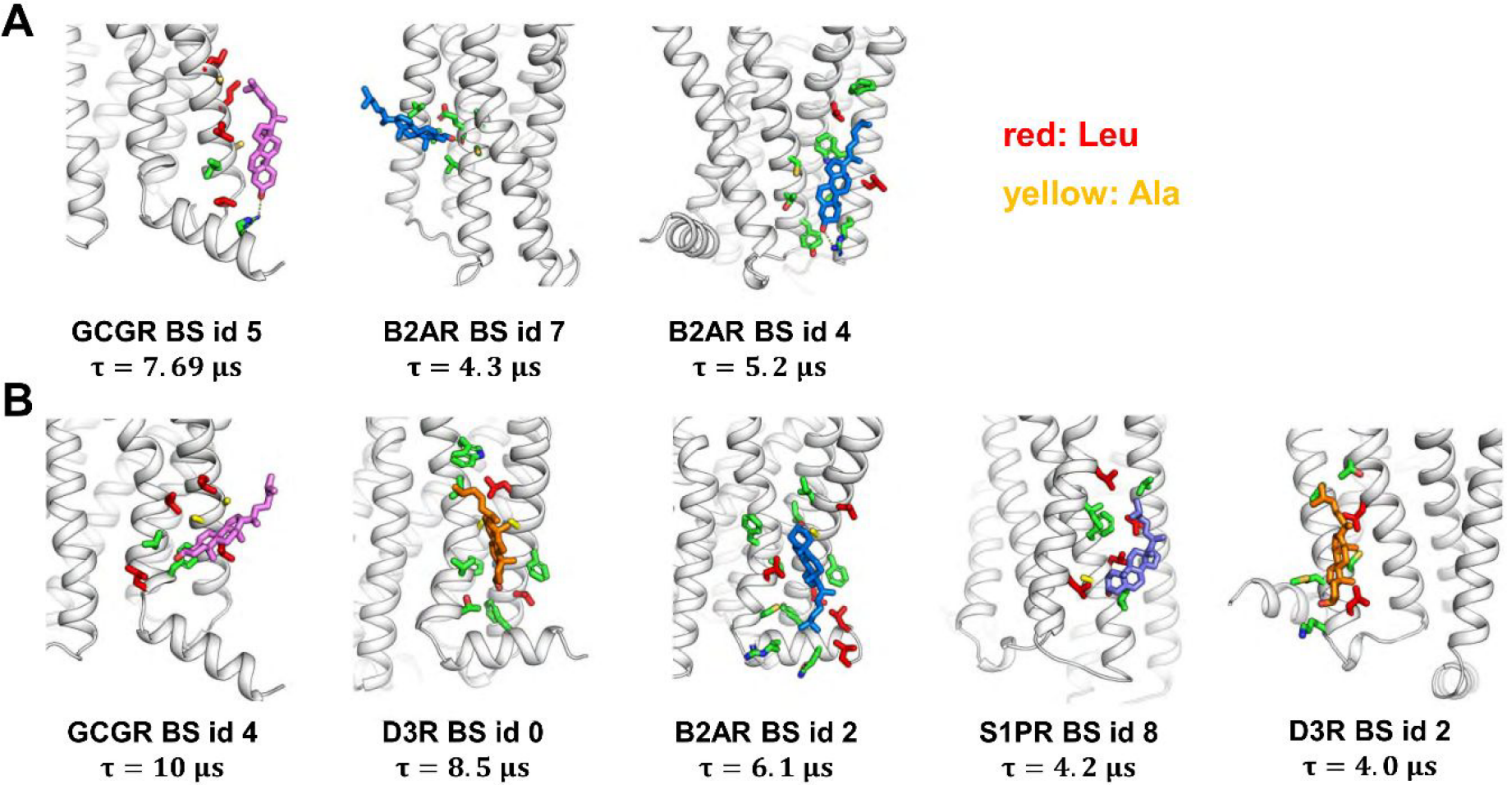
Representative cholesterol bound poses in the eight specific binding sites. (A) Cholesterol bound poses with charge/polar interaction with the hydroxyl group. (B) Cholesterol bound poses without charge–polar interactions. Cholesterols are shown in sticks and colored on the basis of the receptors they are bound to. Protein residues within 0.5 nm of bound cholesterols are shown in green sticks. Text below each bound pose show the receptor name, the binding site (BS) id, and the calculated binding site residence time.

We subsequently examined the representative cholesterol bound poses for these strong binding sites. These bound poses revealed two types of binding modes that are likely to have contributed to the stronger interactions in these binding sites. The first type features polar–charged interactions with the hydroxyl group of cholesterol, as seen in BS (binding site) id 5 of GCGR and at BS id 7 and 4 of β_2_AdR (Figure 6A and SI Figure S14). These polar–charged interactions may be the main stabilizing feature for strong cholesterol binding since the rest of the cholesterol molecule does not show extensive contacts with the receptor in these binding sites. The second type exhibits Leu side chains at the rim of the sites that form a tight grip on the bound cholesterol molecule (Figure 6B and SI Figure S14). These residues might stabilize the cholesterol molecule between the helices.

Taken together, PyLipID has allowed us to analyze cholesterol interactions efficiently and systematically with a set of 10 GPCRs. The analysis of 153 cholesterol binding sites revealed that most cholesterols act as annular lipids around GPCRs, forming transient and potentially nonspecific interactions with the receptors. However, cholesterol may also form longer and more specific interactions with GPCRs at certain binding sites with distinctive structural features. The latter class of sites offer great potential as possible allosteric modulatory sites.

### Two Examples of Characterization of Phospholipid Interactions

We have also explored the application of PyLipID to interactions of membrane proteins with two (anionic) phospholipids, namely, cardiolipin (for bacterial membrane proteins) and PIP_2_ (for mammalian membrane proteins). A recent survey of the energetics of membrane protein–lipid interactions as estimated by MD simulations^28^ has shown that anionic phospholipids interact more strongly with membrane proteins (estimated free energies of −20 to −40 kJ/mol) than is the case for cholesterol (−5 to −10 kJ/mol). Thus, they are expected to exhibit longer residence times and provide good test cases for PyLipID analysis.

We have recently applied PyLipID to analyze cardiolipin interactions for a set of 42 *Escherichia coli* (*E. coli*) inner membrane proteins based on CG-MD simulations using the Martini 3 force field.^62^ Using PyLipID, 700 cardiolipin binding sites were identified, analysis of which yielded a heuristic for defining a high affinity cardiolipin binding site, based on two or three basic residues in proximity, alongside the presence of at least one polar residue and one or more aromatic residues.^28^

**Figure 7:**
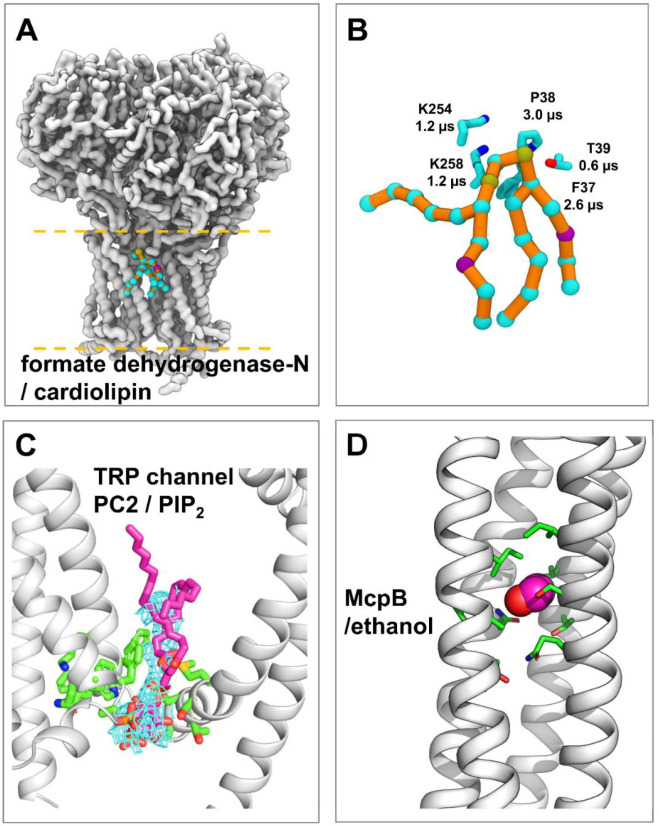
Application of PyLipID to phospholipids and nonlipid molecules. (A) Cardiolipin binding site with the longest residence time on formate dehydrogenase-N. The protein and lipid are described by the Martini CG model. The protein backbone beads are shown in the white surface. The lipid beads are shown in cyan spheres connected by orange sticks. (B) Zoomed-in view of the cardiolipin binding site of formate dehydrogenase-N. The cardiolipin lipid is in the same representation as in panel A. Protein residues that showed the longest residence times in the binding site are shown in sticks. (C) PyLipID calculated PIP_2_ binding site on the TRP channel PC2 overlapping well with the cryo-EM density. The PC2 cryo-EM structure is shown in white cartoon. The PIP_2_ density in the cryo-EM map is shown in blue mesh. The PIP_2_ bound pose calculated by PyLipID is shown in sticks in magenta. The binding site residues calculated by PyLipID are shown in sticks in green. This binding site showed the longest residence time in the Martini CG simulations, as calculated by PyLipID. (D) Ethanol binding sites on McpB. The main ethanol binding sites and an ethanol representative bound pose are shown. McpB is shown in white cartoon and ethanol in spheres. Key side chains are in green.

As an example of this analysis, we have selected formate dehydrogenase-N (PDB id 1KQF), a trimeric membrane protein, each subunit of which has five TM helices and a large cytoplasmic domain. The cardiolipin binding site observed in crystal structure was correctly identified by PyLipID as having the longest residence time among the 16 possible binding sites (Figure 7A and SI Figure S15). Analysis of residence times for individual binding site residues revealed that K254, K258, T39, and the main chain of P38 formed polar interactions or hydrogen bonds with cardiolipin headgroup, contributing to the main stabilizing force for the lipid bound poses in this binding site (Figure 7B). F37 also stabilized the bound lipid by hydrophobic stacking with the lipid tails.

In a second application of PyLipID to anionic lipids, we explored the interaction of PIP_2_ interaction with polycystin-2 (PC2), a TRP channel. A number of studies have implicated PIPs in TRP channel regulations.^12^ On the basis of CG-MD simulations in a membrane containing 10% PIP_2_ in the cytoplasmic leaflet, six binding sites were identified from each of the four subunits of PC2 (SI Figure S6). The PIP_2_ binding site seen in the 3 Å resolution cryo-EM structure (PDB id 6T9N)^44^ was identified by PyLipID as the site with the longest residence time. In addition, the representative bound pose of PIP_2_ in this binding site fits nicely within the lipid-like density in the cryo-EM map (Figure 7C). This again suggests that when multiple possible binding sites are present, residence time analysis using CG-MD simulations and PyLipID can be potentially used to identify the strongest interaction sites corresponding to lipid-like density observed by cryo-EM.

### Application to Interactions of a Nonlipid Ligand with a Membrane Protein

PyLipID can be readily applied to characterize the binding of nonlipid molecules in conjunction with atomistic simulations whenever sufficient binding/unbinding events are sampled. It therefore may be particularly useful for, e.g., fragment screening approaches to binding site discovery^64^ (in particular, see ref (65) for an early application of this approach to GPCRs and ref (66) for a recent application using Martini 3).

To demonstrate the application of PyLipID to small molecule/fragment binding, we analyzed the interactions of ethanol with a bacterial chemoreceptor, McpB, for which ethanol is a known attractant. The analyses were carried out on previously conducted atomistic simulations (3 × 600 ns) of an McpB cytoplasmic homodimer with 165 ethanol molecules (0.316 M) included to reproduce experimental conditions.^52^ As anticipated, ethanol molecules showed transient interactions with the receptor due to their small size and simple structure. Using PyLipID a total of 50 ethanol binding sites were identified on McpB, with residence times ranging from sub-nanosecond to ∼40 ns (SI Figure S16). Notably, the analysis highlighted several binding sites with longer residence times located within the center of the coiled-coil bundle (Figure 7D). It is suggested that these may facilitate conformational changes induced by ethanol binding to be transmitted to other parts of the receptor, thereby enabling the signaling response. To test the sensitivity of PyLipID to minor changes in protein sequence, we additionally analyzed atomistic simulations (3 × 600 ns) of McpB carrying the A431S mutation, which is known to considerably reduce taxis to alcohols.^52^ While the 51 ethanol binding sites identified by PyLipID largely overlap with those on wild-type McpB, ethanol binding to the side chain of residue 431 was no longer observed (SI Figure S16). This example suggests therefore that PyLipID could be usefully employed as an analysis tool within an MD-based fragment screening study.

## Discussion and Conclusions

### What Does PyLipID Allow Us to Do?

We have described PyLipID, an integrated package for analysis of protein–lipid interactions from MD simulation data. PyLipID has the following main features:

1. It calculates binding sites from simulation data using a robust methodology.
2. It calculates the residence times for lipid interactions with both the binding sites and individual amino acid residues.
3. It generates bound lipid poses and outputs structural representatives for each binding site.
4. It uses a dual-cutoff scheme to robustly quantify lipid interactions in a manner suitable for dynamic interactions in both coarse-grained and atomistic simulations.
5. It outputs interaction data in a convenient format to assist the ease and customization of subsequent large scale data analysis.

Thus, PyLipID provides for systematic and standardized analysis of protein–lipid interactions over large simulation data sets from multiple membrane proteins, facilitating comparative analysis of lipid binding sites. The inclusion of functions to generate representative bound poses allows for in-depth analysis alongside experimental structural data. PyLipID is an open-source Python package which allows users to customize the functions. It provides various portals for further manipulation of the generated data. It can be readily incorporated into analysis scripts, allowing for high throughput analysis of big data sets.^62^

### How Does PyLipID Compare with Other Software in This Area?

There are several frameworks developed for analysis of membrane MD simulations, building on the considerable expansion in this area of research over recent years. The closest in spirit to PyLipID is ProLint.^67^ ProLint is Web-based, but also available as a standalone Python package *prolintpy*. ProLint provides feature-rich visualization and analysis tools, leaving binding site interpretations up to the user. In this respect it differs from PyLipID which automatically defines and analyses lipid binding sites to facilitate comparison with experiments and to provide more directly pharmaceutically relevant structural insights. A somewhat simpler membrane protein simulation analysis framework is provided by MemProtMD,^68^ a database of CG-MD simulations of all known membrane protein structures in a model bilayer, which provides contact-based metrics for protein–lipid interactions, and information local bilayer thickness distortion by proteins. MemProtMD is now directly linked to membrane protein entries by RCSB/PDB. There have also been several recent packages developed which are aimed at analysis of lipid bilayers. These include, e.g., LiPyphilic,^69^ which is a fast Python package for analyzing complex lipid bilayer simulations (but not yet extended to membrane proteins), and FATSLiM,^70^ also in Python, which enables bilayer leaflet identification and bilayer thickness and area per lipid calculations, and which works for various (curved) membrane geometries and bilayers including proteins. In terms of more detailed analysis of interactions at binding sites, there are several more general approaches for drug-target residence times via simulations, including, e.g., τRAMD,^71^ which may in principle be adaptable to protein–lipid interactions.

### What Can PyLipID Teach Us about Protein-Lipid Interactions?

We have described a couple of applications of PyLipID. There is considerable literature on identifying and characterizing GPCR–cholesterol interactions by MD simulations (e.g., see refs (32, 72, and 73)), and it is not our aim to review these here (for recent reviews see, e.g., refs (12 and 74)). There have also been a number of GPCR structural studies, e.g., combined with docking of cholesterol^75^ to generate a database of predicted binding sites for cholesterol on membrane proteins, or via analysis of crystal structures of GPCRs with bound cholesterol molecules.^61^ PyLipID provides some new insights into GPCR–cholesterol interactions. In particular, the analysis of residence times has allowed us to separate interactions/sites in annular and specific cholesterol binding sites, the latter showing longer residence times and having enriched interactions with Leu, Gly, and Ala residues. Extending this approach to a couple of anionic phospholipids suggests that long residence time binding sites correlate with those observed experimentally in cryo-EM structures, indicating how PyLipID may be used to aid the assignment and analysis of lipid-like density in newly determined structures.^76^

## Supporting Information Available

(Figures S1–S16) PyLipID package design, minimum distances and distributions, various calculations, binding site characterizations, and residence time plots; choice and impacts of cutoffs and additional methods details; table listing simulation overview (PDF)
